# The Impact of Weight Categories on the Association Between Atrial Fibrillation/Flutter and Known Risk Factors: A Nationwide Inpatient Data Analysis

**DOI:** 10.3390/jcm14072187

**Published:** 2025-03-23

**Authors:** Kennedy Sparling, Mehrtash Hashemzadeh, Mohammad Reza Movahed

**Affiliations:** 1 Department of Medicine, University of Arizona, Phoenix, AZ 85054, USA; ksparling@arizona.edu (K.S.); mehrtash2001@yahoo.com (M.H.); 2 Department of Medicine, University of Arizona Sarver Heart Center, Tucson, AZ 85719, USA

**Keywords:** obesity paradox, obesity, atrial fibrillation, atrial flutter, morbid obesity, risk factors, arrhythmia

## Abstract

**Background/Objectives**: Atrial fibrillation and atrial flutter (Afib/Aflut) are the most common arrhythmias presenting to the emergency department. The goal of this study was to evaluate any predictor of Afib/flut with cardiovascular risk factors and demographics based on weight categories. **Methods**: Using ICD-10 codes from the large Nationwide Inpatient Sample (NIS) database in the years 2016–2020, we evaluate any association between the presence of Afib/Aflut with risk factors and demographics in different weight categories in adults over the age of 18. **Results**: A total of 23,037,013 afib/flut patients were found in the NIS database. Obesity and morbid obesity were independently associated with the presence of Afib/Aflut (for multivariate OR obesity: 1.28, CI 1.27–1.28, *p* < 0.001; for morbid obesity: OR 1.9, CI 1.89–1.91, *p* < 0.001). Regardless of weight categories such as cachexia, overweight, obese, or morbidly obese, traditional risk factors remained independently associated with Afib/Aflut. Furthermore, male gender and Caucasians were independently associated with the presence of Afib/Aflut regardless of any weight categories. (For example, in the overweight categories, the multivariate OR for females was 0.69, CI: 0.69–0.69, *p* < 0.001, and for African Americans, OR 0.62, CI 0.61–0.62, *p* < 0.001). **Conclusions**: Traditional risk factors were persistently associated with the occurrence of atrial fibrillation regardless of weight categories. Furthermore, the Caucasian race and male gender were also strong independent predictors of Afib/Aflut.

## 1. Introduction

**Figure 1:**
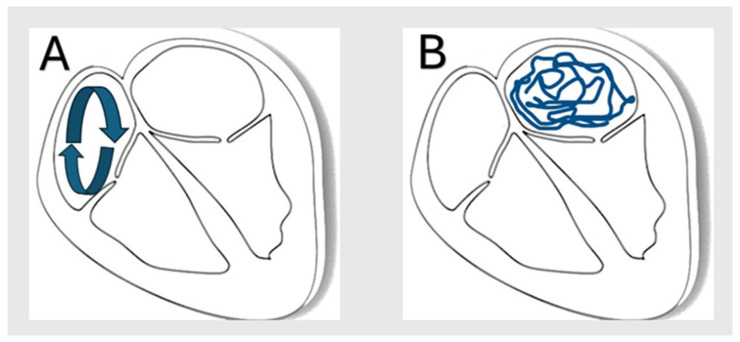
(**A**) mechanism of atrial flutter showing organized re-entry in the right atrium, (**B**) showing disorganized micro re-entry in the atrial muscle originating mostly from left atrium.

Atrial fibrillation (Afib) and atrial flutter (Aflut) are among the most common arrhythmias, with over 4 million cases reported annually [1,2]. Both Afib and Aflut are classified as supraventricular tachyarrhythmias; however, they exhibit distinct electrophysiological differences. Afib is recognized as uncoordinated atrial activation due to electrical input from an ectopic focus (Figure 1A), while Aflut is due to rapid, organized atrial activity secondary to electrical re-entry (Figure 1B) [3]. Due to the sequelae of events that can occur when a patient experiences Afib or Aflut, these cardiac conditions present serious public health risks with considerable morbidity and mortality. In 2021 alone, Afib and Aflut were associated with about 8.38 million disability-adjusted life years globally [2]. Therefore, deepening our understanding of these arrhythmias is essential to improving patient outcomes and dampening their public health burden.

By understanding the demographics and risk factors associated with Afib and Aflut, the medical community can work toward more effective prevention strategies. Established risk factors include obesity, sedentary lifestyle, diabetes mellitus (DM), smoking, hypertension (HTN), chronic kidney disease (CKD), Myocardial Infarctions (MIs), and chronic obstructive pulmonary disease (COPD) [4,5,6,7,8,9,10]. In fact, prior studies have revealed that HTN is the most common risk factor for these arrhythmias, with over 60% of patients with Afib being affected by both conditions. Fortunately, some of these factors can be prevented or reversed, emphasizing the importance of primary and secondary prevention with Afib and Aflut [11,12]. On the other hand, while demographic characteristics associated with Afib and Aflut, including being of the male sex and the Caucasian race, cannot be modified, understanding populations at a higher risk can allow for improved risk stratification and facilitate the closer monitoring of these patients [13].

Although obesity is a known risk factor for Afib and Aflut, the other mentioned factors also share a relationship with obesity. This prompts the question of whether the degree of these associations with Afib or Aflut hold true regardless of weight category. Prior studies have revealed that these risk factors, including HTN, DM, MI, and COPD, all serve as independent risk factors for Afib even after multivariable adjustment [14,15]. However, understanding how these factors interact with varying levels of weight can yield valuable insights into their combined effects. As the odds of developing Afib or Aflut increase by approximately 30% with every five-point BMI increase, it is crucial to consider how specific weight categories, including cachexic, overweight, obesity, and morbid obesity, interact with known risk factors to modify this likelihood [16,17].

Therefore, this large retrospective cohort study aimed to evaluate possible predictors of Afib and Aflut using known risk factors and demographics categorized by weight categories. Our overarching goal is to determine which risk factors share the strongest association with Afib and Aflut for each weight category. In doing so, this study can offer guidance for developing tailored prevention plans by identifying whether different targets should be prioritized based on a patient’s weight.

## 2. Methods

### 2.1. Data Source

This study employed data from the National Inpatient Sample (NIS) database, which is one of the largest inpatient datasets offered by the Agency for Healthcare Research and Quality [18]. This database incorporates information from a 20% sample of hospital discharges throughout the United States [18]. As this resource does not contain any patient-identifying information and is publicly accessible, this study did not require institutional review board approval.

### 2.2. Study Population

The NIS database was analyzed for patients aged 18 and older with a prior diagnosis of Afib or Aflut from 2016 to 2020. Data from the years 2016 to 2020 were collected. These patients were identified using the International Classification of Diseases, 10th revision (ICD-10) codes for Afib and Aflut (Table 1).

### 2.3. Study Outcome and Statistical Analysis

This study aimed to evaluate the association between an Afib or Aflut diagnosis with various risk factors and demographics across the various weight categories in adults. These factors were incorporated into a multivariate analysis when evaluating the relationship between Afib or Aflut and weight category. These variables were extracted from the NIS database using their corresponding ICD-10 code, as outlined in Table 1.

Patient demographic, clinical, and hospital characteristics were reported as the median (IQR) for continuous variables and proportions and 95% confidence intervals for categorical variables. Logistic regression was performed to ascertain the odds of binary clinical outcomes relative to patient and hospital characteristics as well as the odds of clinical outcomes over time. All statistical models were adjusted for confounding. All analyses were conducted following the implementation of population discharge weights. All *p*-values were 2-sided, and *p* < 0.05 was considered statistically significant. The data were analyzed using STATA 17 (Stata Corporation, College Station, TX, USA).

## 3. Results

A total of 23,037,013 patients with Afib or Aflut from the NIS database were identified. The median age of the participants was 77 years, with an interquartile range of 67 to 84 years. Forty-seven percent of the participants were female. Moreover, 78.27% of the participants were of the white race.

**Figure 2:**
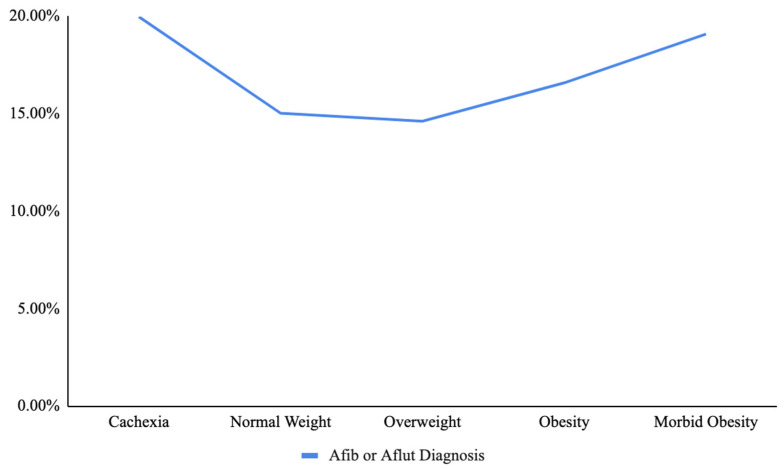
Percent of patients with a diagnosis of atrial fibrillation (Afib) or atrial flutter (Aflut) in each weight category.

The relationship between weight category and a diagnosis of Afib or Aflut was assessed, incorporating traditional risk factors and demographic characteristics into a multivariate analysis. Patients diagnosed with Afib or Aflut had significantly higher odds of being classified as obese (multivariate odds ratio (OR): 1.28, CI 1.27–1.28, *p* < 0.001) and morbidly obese (multivariate OR: 1.9, CI 1.89–1.91, *p* < 0.001) compared to those with a normal weight (Figure 1). Further, patients diagnosed with Afib or Aflut also displayed marginally higher odds of being classified as cachexic (multivariate OR for cachexia: 1.02, CI 1.01–1.03, *p* = 0.002). On the other hand, patients diagnosed with Afib or Aflut exhibited slightly lower odds of being classified as overweight (multivariate OR for overweight: 0.96, CI 0.93–0.99, *p* = 0.005) (Figure 2).

**Figure 3:**
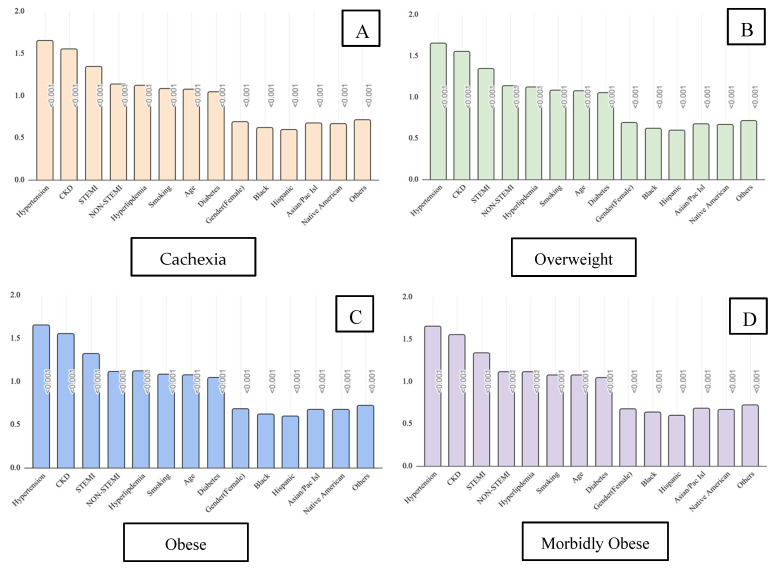
The Odds ratio of a diagnosis of atrial fibrillation (Afib) or atrial flutter (Aflut) for known cardiac risk factors and demographics in each weight category, including cachexic (**A**), overweight (**B**), obese (**C**), and morbidly obese (**D**).

Moreover, traditional risk factors associated with Afib and Aflut, including smoking, diabetes mellitus (DM), hypertension (HTN), chronic kidney disease (CKD), hyperlipidemia (HLD), ST-Elevation Myocardial Infarction (STEMI), and Non-ST-Elevation Myocardial Infarction (non-STEMI), were independently associated with an Afib or Aflut diagnosis, with similar ORs irrespective of weight category (Table 2; Figure 3). Furthermore, the male gender and white race were also independently associated with the presence of Afib or Aflut across all weight categories (Table 2).

## 4. Discussion

The objective of this study was to identify potential predictors of AFib and AFlut through an analysis of known risk factors and demographics, categorized by weight groups. This investigation revealed that higher levels of obesity significantly increased the odds of developing AFib or AFlut. Additionally, it indicated a slightly reduced risk for those categorized as overweight, while individuals classified as cachexic exhibited a marginally elevated risk for these arrhythmias. These findings suggest that the association between one’s weight and the risk of Afib and Aflut is complex and not as linear nor exponentially increasing as prior studies have suggested [19,20]. Further, it highlights that a low BMI may pose unique challenges that contribute to one’s risk of Afib or Aflut, which is a finding that has varied in prior studies [21,22,23].

Moreover, this study highlighted that traditional risk factors continued to be associated with Afib and Aflut regardless of weight. Specifically, HTN was consistently the strongest risk factor across all weight categories, followed by CKD, STEMI, non-STEMI, and HLD. This suggests that prevention plans should prioritize strict blood pressure control for all patients regardless of their BMI. The other risk factors should also be addressed for all patients, regardless of their weight. While previous studies have exhibited this same trend, they did not reveal how BMI is not a substantial factor in altering the influence these comorbidities have on Afib and Aflut [24,25,26,27].

Although not completely elucidated, it is thought that weight affects the likelihood of developing Afib or Aflut in multiple ways, including through systemic inflammation, cardiac remodeling, and adipose deposition [28,29]. Similarly, HTN and MIs are thought to alter cardiac structure and electrical conduction, while COPD has been suggested to contribute through alterations in blood gas levels and cardiac remodeling secondary to pulmonary HTN [5,11,30,31,32]. Additionally, CKD is thought to increase one’s risk of Afib and Aflut due to electrolyte and blood cell abnormalities as well as the activation of the renin–angiotensin–aldosterone system (RAAS) [33,34,35]. Although these factors are thought to increase one’s likelihood of developing these arrhythmias through similar mechanisms, our study highlights that weight neither protects nor exacerbates the influence of these risk factors on the development of Afib or Aflut. Furthermore, although chronic inflammation contributes to the development of Afib or Aflut in all of these chronic conditions, it is noteworthy that the effects of inflammation most likely do not compound or intensify one another [11,29,30,32,35].

Lastly, this investigation displayed that the Caucasian race and male gender were strong independent predictors of Afib and Aflut. This similar finding has been shown in previous studies, highlighting the role that biological influences play in the development of these arrhythmias [36,37,38]. This relationship is thought to be due to polymorphisms in the RAAS, ion channel, and signaling molecule genes that contribute to alterations in cardiac structural and electrical function [38,39]. Although other races display a higher prevalence of the aforementioned risk factors, the Caucasian race continues to be a strong predictor of these arrhythmias, suggesting that genetic influences may outweigh environmental ones when it comes to Afib and Aflut. While these factors cannot be modified, clinicians should be aware of them to effectively stratify risk in patients and identify those at increased odds of developing these arrhythmias.

While our study displayed that traditional risk factors were persistently associated with Afib or Aflut regardless of weight category, future research should focus on the impact of weight categories on treatments and prevention strategies of these arrhythmias. Prior investigations have noted that weight can affect anti-arrhythmic and anticoagulant drug properties, worsen outcomes after ablation, and alter the underlying chemical mediators of Afib development [40,41]. This suggests that, although weight may not directly influence the effect of risk factors on Afib or Aflut development, interventions may lead to different outcomes depending on one’s weight category. This notion underscores the continued importance of a further exploration into the role weight category plays in Afib and Aflut development, prevention, and management. Furthermore, it emphasizes the importance of a multidisciplinary approach to caring for patients with Afib or Aflut, given the role nutrition may play in weight and subsequent outcomes.

## 5. Conclusions

Overall, this study highlighted how traditional cardiac risk factors, including HTN, DM, CKD, HLD, STEMI/Non-STEMI, and smoking, were persistently associated with the occurrence of atrial fibrillation regardless of BMI. As odd ratios remained relatively consistent across all weight categories and varying weight categories differentially impact one’s risk of Afib or Aflut, our study suggests that the relationship between weight and these arrhythmias is multifaceted and not necessarily as linear nor compounding as prior studies have suggested [19,20]. Consequently, further studies should focus on the role of weight in the prevention and management of Afib and Aflut. Moreover, an additional exploration into the utility of bioimpedance analysis, the association between B-type natriuretic peptide and obesity, and the role of ultrasound and computed tomography in evaluating visceral fat could further enhance our understanding of the impact of weight on Afib and Aflut.

### Limitations

This study possesses various limitations that may affect our findings. As noted previously, the NIS database only contains one-fifth of hospital discharges throughout America [18]. This may introduce a potential bias due to the incomplete representation of all discharges and may also diminish generalizability beyond the U.S. demographic. Furthermore, as this database only contains information from the inpatient population, trends in outpatient settings could not be assessed. Nonetheless, the very large sample size enables this study to provide valuable insight into the relationship that weight has with other influencing factors in the development of Afib and Aflut. Additionally, there may be additional lifestyle factors that can influence the development of Afib and Aflut that were not included in this study. These include dietary factors as well as alcohol and caffeine consumption [42]. Lastly, this study was retrospective in nature, which means that a direct causal relationship could not be established.

## Figures and Tables

**Table 1:** The International Classification of Diseases, 10th revision (ICD-10) codes for the variables evaluated in this study.

Variable	ICD-10 Code(s)
Atrial Fibrillation and Flutter	I48
Cachexia	R64
Overweight	E66.3
Obesity	E66.9, E66.8, E66.0
Morbid Obesity	E66.01, E66.2
Smoking	F17.20, Z72.0, Z87.891
Diabetes	E08–E13
Hypertension	I10, I11.0, I11.9, I120, I129, I13.0, I13.10, I13.11, I13.2, I15.0, I15.1, I15.2, I15.9, I16.0, I16.1, I16.9
Chronic Obstructive Pulmonary Disease	J41.0, J41.1, J41.8, J42., J43.0, J43.1, J43.2, J43.8, J43.9, J44.0, J44.1, J44.9, J47.0, J47.1, J47.9, J684
Chronic Kidney Disease	I13.11, I13.2, N289, Q613, N181, N182, N183, N1830, N1831, N1832, N184, N185, N186, N189, R880, N19
ST-Elevation Myocardial Infarction	I21.01, I21.02, I21.09, I21.11, I21.19, I21.21, I21.29, I21.3, I21.9, I21.A1, I21.A9, I22.0, I22.1, I22.5, I22.9
Non-ST-Elevation Myocardial Infarction	I21.4, I22.2
Old Myocardial Infarction	I25.2

**Table 2:** The association between a diagnosis of atrial fibrillation or atrial flutter and their traditional risk factors, including smoking, diabetes mellitus (DM), hypertension (HTN), chronic kidney disease (CKD), hyperlipidemia (HLD), ST-Elevation Myocardial Infarction (STEMI), and Non-ST-Elevation Myocardial Infarction (non-STEMI), as well as demographic characteristics across weight categories. The confidence interval (CI) for the odds ratios (OR) is 95%.

Variable	Cachexia		Overweight		Obesity		Morbid Obesity	
	OR	CI	OR	CI	OR	CI	OR	CI
Smoking	1.08	1.07–1.08	1.08	1.08–1.09	1.08	1.07–1.08	1.07	1.07–1.08
DM	1.04	1.04–1.05	1.05	1.04–1.05	1.04	1.04–1.04	1.04	1.04–1.04
HTN	1.65	1.64–1.66	1.65	1.64–1.66	1.65	1.64–1.66	1.65	1.64–1.66
CKD	1.55	1.54–1.55	1.55	1.54–1.55	1.55	1.54–1.55	1.55	1.54–1.55
HLD	1.12	1.12–1.13	1.12	1.12–1.12	1.12	1.11–1.12	1.11	1.1–1.11
STEMI	1.34	1.33–1.36	1.34	1.33–1.36	1.32	1.31–1.34	1.33	1.32–1.35
Non-STEMI	1.13	1.12–1.14	1.13	1.12–1.14	1.11	1.1–1.12	1.11	1.1–1.12
Age	1.07	1.07–1.07	1.07	1.07–1.07	1.07	1.07–1.07	1.07	1.07–1.07
Gender (Female)	0.69	0.69–0.69	0.69	0.69–0.69	0.68	0.68–0.68	0.67	0.67–0.68
Race (Black)	0.62	0.61–0.62	0.62	0.61–0.62	0.62	0.62–0.63	0.63	0.62–0.63
Race (Hispanic)	0.59	0.58–0.59	0.59	0.58–0.59	0.59	0.58–0.59	0.59	0.58–0.59
Race (Asian/Pacific Islander)	0.67	0.66–0.68	0.67	0.66–0.68	0.67	0.66–0.68	0.68	0.67–0.69
Race (Native American)	0.66	0.64–0.69	0.66	0.64–0.69	0.67	0.64–0.69	0.66	0.64–0.69
Race (Other)	0.71	0.7–0.73	0.71	0.70–0.73	0.72	0.7–0.73	0.72	0.7–0.73

## Data Availability

The original contributions presented in this study are included in the article. Further inquiries can be directed to the corresponding author.
